# Supplementary figures and images for: Analysis of English free association network reveals mechanisms of efficient solution of Remote Association Tests
Source: PLoS One. 2021 Apr 6;16(4):e0248986. doi: 10.1371/journal.pone.0248986 (PMC8023469; doi:10.1371/journal.pone.0248986)

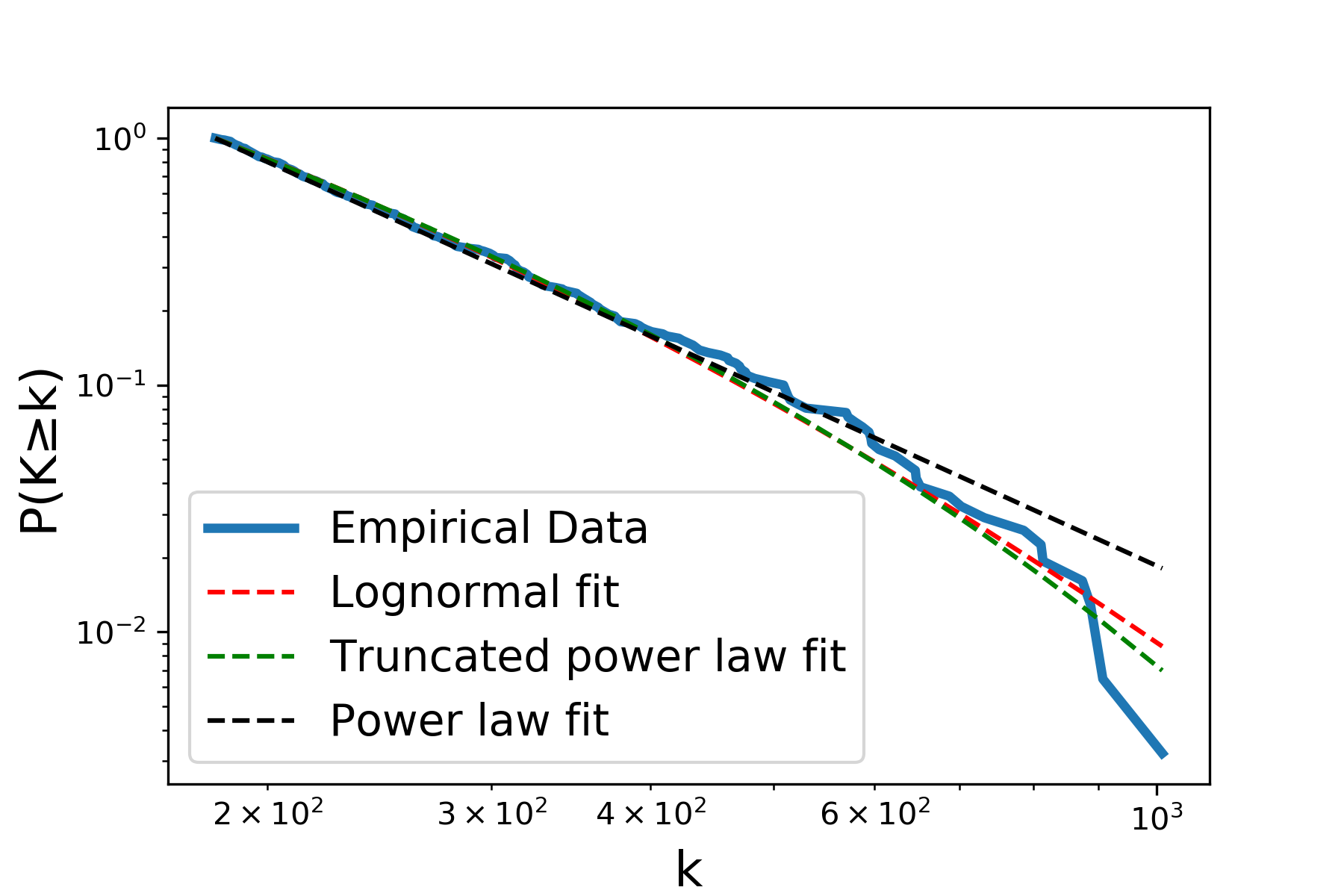

Supplement: S1 Fig — We compared the fits of three candidate distribution using Python package powerlaw [40], results of likelihood-ratio test are presented in S1 Fig. (TIF) [file pone.0248986.s001.tif]

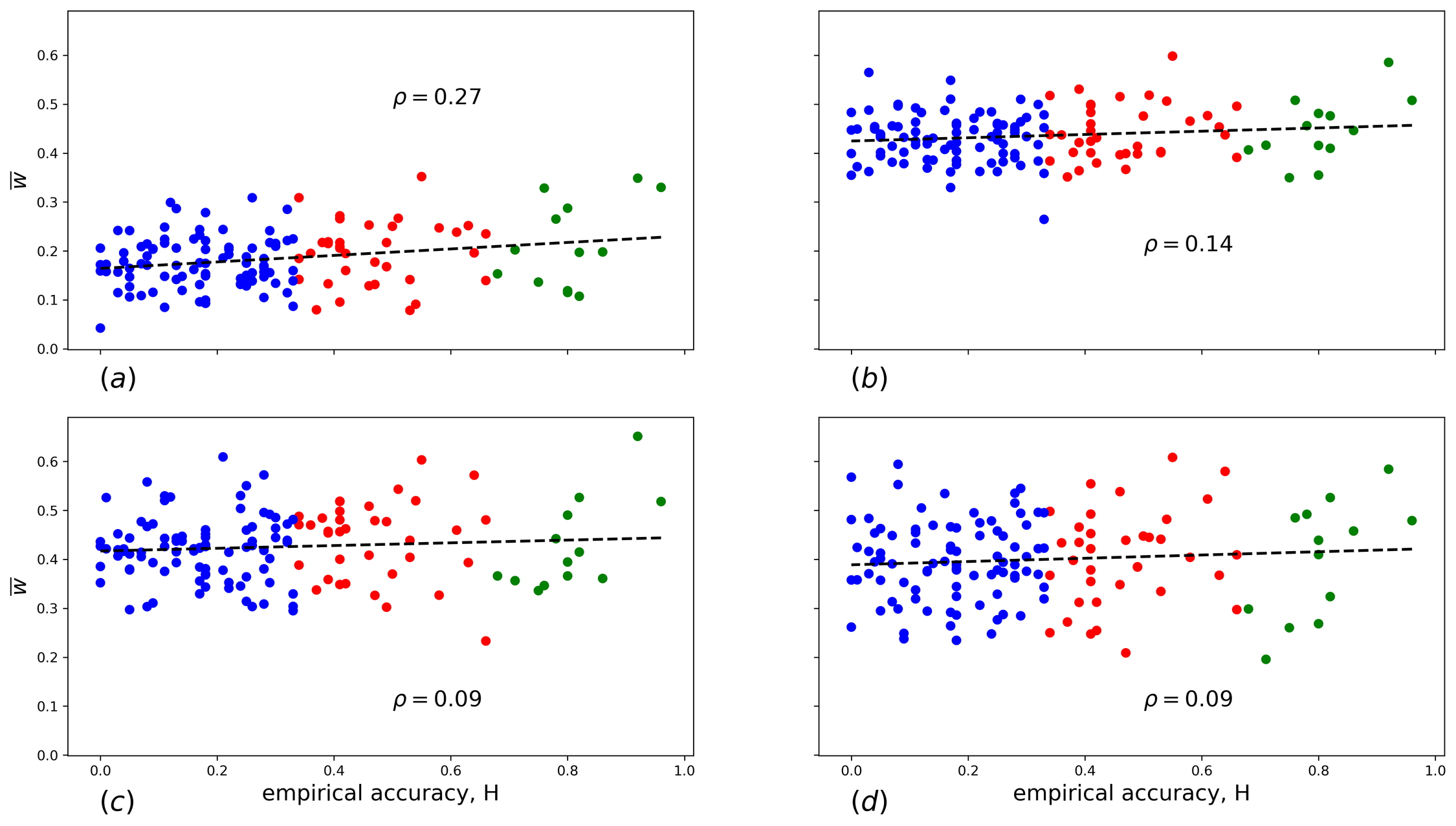

Supplement: S2 Fig — We used pre-trained vector representations for words from different model: (a) word2vec GoogleNewsvectors [9]; (b) Fast Text Wiki News [41]; (c) Glove Wikipedia + Gigaword [42]; (d) Glove Twitter [42]. In all figures ρ is the Pearson correlation coefficient. (TIF) [file pone.0248986.s002.tif]
