# Supplementary material for: Analysis of English free association network reveals mechanisms of efficient solution of Remote Association Tests
Source: PLoS One. 2021 Apr 6;16(4):e0248986. doi: 10.1371/journal.pone.0248986 (PMC8023469; doi:10.1371/journal.pone.0248986)
Supplement: S1 Table — The best fit to truncated power law p(k) = k−α e−βk is established with the respective parameters: α = 2.3, β = 0.002. (PDF) [file pone.0248986.s003.pdf]

Easy RATs		
Remote Associate Stimuli	Solution	% of pers. solving RAT in 15 sec
cottage, Swiss, cake	cheese	96
cream, skate, water	ice	92
loser, throat, spot	sore	86
show, life, row	boat	82
night, wrist, stop	watch	82
duck, fold, dollar	bill	80
rocking, wheel, high	chair	80
dew, comb, bee	honey	80
fountain, baking, pop	soda	78
preserve, ranger, tropical	forest	76
aid, rubber, wagon	band	75
flake, mobile, cone	snow	71
cracker, fly,fighter	fire	68
safety, cushion, point	pin	66
cane, daddy, plum	sugar	66

Medium RATs		
Remote Associate Stimuli	Solution	% of pers. solving RAT in 15 sec
dream, break, light	day	64
fish, mine, rush	gold	63
political, surprise, line	party	61
measure, worm, video	tape	58
high, district, house	school	55
sense, courtesy, place	common	54
worm, shelf, end	book	53
piece, mind, dating	game	53
flower, friend, scout	girl	51
river, note, account	bank	50
print, berry, bird	blue	49
pie, luck, belly	pot	49
date, alley, fold	blind	47
opera, hand, dish	soap	47
fur, rack, tail	coat	46
stick, maker, point	match	46
hound, pressure, shot	blood	42
fox, man, peep	hole	42
sleeping, bean, trash	bag	41
dust, cereal, fish	bowl	41
light, birthday, stick	candle	41
food, forward, break	fast	41
peach, arm, tar	pit	41
water, mine, shaker	salt	41
palm, shoe, house	tree	41
basket, eight, snow	ball	39
wheel, hand, shopping	cart	39
right, cat, carbon	copy	39
home, sea, bed	sick	38
nuclear, feud, album	family	37
sandwich, house, golf	club	36
cross, rain, tie	bow	34
sage, paint, hair	brush	34
French, car, shoe	horn	34
boot, summer, ground	camp	33
chamber, mask, natural	gas	33
mill, tooth, dust	saw	33
pike, coat, signal	turn	33
office, mail, hat	box	32
fly, clip, wall	paper	32
age, mile, sand	stone	32
