# Supplementary material for: Analysis of English free association network reveals mechanisms of efficient solution of Remote Association Tests
Source: PLoS One. 2021 Apr 6;16(4):e0248986. doi: 10.1371/journal.pone.0248986 (PMC8023469; doi:10.1371/journal.pone.0248986)
Supplement: S2 Table — The list of 138 RATs we have used in our research and their hardness according to the data of [34]. (PDF) [file pone.0248986.s004.pdf]

| Hard RATs (part I) | | |
|---|---|---|
| Remote Associate Stimuli | Solution | % of pers. solving RAT in 15 sec |
| catcher, food, hot | dog | 30 |
| wagon, break, radio | station | 30 |
| tank, hill, secret | top | 30 |
| health, taker, less | care | 29 |
| lift, card, mask | face | 29 |
| dress, dial, flower | sun | 29 |
| force, line, mail | air | 28 |
| guy, rain, down | fall | 28 |
| eight, skate, stick | figure | 28 |
| down, question, check | mark | 28 |
| animal, back, rat | pack | 28 |
| officer, cash, larceny | petty | 28 |
| pine, crab, sauce | apple | 26 |
| house, thumb, pepper | green | 26 |
| carpet, alert, ink | red | 26 |
| master, toss, finger | ring | 26 |
| hammer, gear, hunter | head | 25 |
| knife, light, pal | pen | 25 |
| foul, ground, mate | play | 25 |
| change, circuit, cake | short | 25 |
| way, board, sleep | walk | 25 |
| blank, list, mate | check | 24 |
| tail, water, flood | gate | 24 |
| cover, arm, wear | under | 24 |
| rain, test, stomach | acid | 22 |
| pile, market, room | stock | 22 |
| mouse, bear, sand | trap | 22 |
| cat, number, phone | call | 21 |
| keg, puff, room | powder | 21 |
| trip, house, goal | field | 18 |
| fork, dark, man | pitch | 18 |
| fence, card, master | post | 18 |
| test, runner, map | road | 18 |
| dive, light, rocket | sky | 18 |
| man, glue, star | super | 18 |
| tooth, potato, heart | sweet | 18 |
| illness, bus, computer | terminal | 18 |
| type, ghost, screen | writer | 18 |
| mail, board, lung | black | 17 |
| teeth, arrest, start | false | 17 |
| iron, shovel, engine | steam | 17 |

| Hard RATs (part II) | | |
|---|---|---|
| Remote Associate Stimuli | Solution | % of pers. solving RAT in 15 sec |
| wet, law, business | suit | 17 |
| rope, truck, line | tow | 17 |
| off, military, first | base | 16 |
| spoon, cloth, card | table | 16 |
| cut, cream, war | cold | 14 |
| note, chain, master | key | 14 |
| shock, shave, taste | after | 13 |
| wise, work, tower | clock | 13 |
| grass, king, meat | crab | 13 |
| baby, spring, cap | shower | 13 |
| break, bean, cake | coffee | 12 |
| cry, front, ship | battle | 11 |
| hold, print, stool | foot | 11 |
| roll, bean, fish | jelly | 11 |
| horse, human, drag | race | 11 |
| oil, bar, tuna | salad | 11 |
| bottom, curve, hop | bell | 9 |
| pea, shell, chest | nut | 9 |
| line, fruit, drunk | punch | 9 |
| bump, egg, step | goose | 8 |
| fight, control, machine | gun | 8 |
| home, arm, room | rest | 8 |
| child, scan, wash | brain | 7 |
| nose, stone, bear | brown | 7 |
| end, line, lock | dead | 7 |
| control, place, rat | birth | 5 |
| lounge, hour, napkin | cocktail | 5 |
| artist, hatch, route | escape | 5 |
| pet, bottom, garden | rock | 5 |
| mate, shoes, total | running | 5 |
| self, attorney, spending | defense | 4 |
| board, blade, back | switch | 4 |
| land, hand, house | farm | 3 |
| hungry, order, belt | money | 3 |
| forward, flush, razor | straight | 3 |
| shadow, chart, drop | eye | 1 |
| way, ground, weather | fair | 1 |
| cast, side, jump | broad | 0 |
| back, step, screen | door | 0 |
| reading, service, stick | lip | 0 |
| over, plant, horse | power | 0 |
