# Supplementary material for: Analysis of English free association network reveals mechanisms of efficient solution of Remote Association Tests
Source: PLoS One. 2021 Apr 6;16(4):e0248986. doi: 10.1371/journal.pone.0248986 (PMC8023469; doi:10.1371/journal.pone.0248986)
Supplement: S1 Text — (PDF) [file pone.0248986.s005.pdf]

S1 Text.

Word embeddings are vector representations of a word obtained by training a neural network on a large corpus. Word2vec is one of the most widely used forms of word embeddings. The word2vec takes text corpus as input and produces word vectors as output, which can be further used to train any other word to obtain its corresponding vector value. This word2vec model uses a continuous skip-gram model [1], based on the distributional hypothesis. An open-source library, Gensim [2] provides different pre-trained models based on different machine learning algorithms (GloVe, ConceptNet) and different kinds of datasets like googlenews, Wikipedia, twitter. These pre-trained word embeddings was used to create feature vectors for the dataset in this experiment. We calculated the average similarity from the stimuli words to the response according simple strategy (1). Weak correlations shows that not only semantic nature should be considered in simulation of the RAT solving.

References

1. T. Mikolov, I. Sutskever, K. Chen, G. Corrado, and J. Dean, Distributed Representations of Words and Phrases and their Compositionality, Advances in Neural Information Processing Systems 26 (NIPS 2013), arXiv:1310.4546 (2013).

2. R. Rehurek and P. Sojka, Software Framework for Topic Modelling with Large Corpora, Proceedings of the LREC 2010 Workshop on New Challenges for NLP Frameworks, pp. 45-50 (2010).
