# Supplementary material for: Analysis of English free association network reveals mechanisms of efficient solution of Remote Association Tests
Source: PLoS One. 2021 Apr 6;16(4):e0248986. doi: 10.1371/journal.pone.0248986 (PMC8023469; doi:10.1371/journal.pone.0248986)
Supplement: Attachment — Submitted filename: Response to Reviewers.pdf [file pone.0248986.s006.pdf]

Response to the Reviewer's Comments

Olga Valba, Alexander Gorsky, Sergei Nechaev, Mikhail Tamm

February 19, 2021

1 Journal Requirements

Please ensure that your manuscript meets PLOS ONE's style requirements, including those for file naming.

We have changed affiliations and renamed files according to the requirements of the journal.

2 Reviewers' comments

Reviewer #1: PONE-D-20-34630
Analysis of English free association network reveals mechanisms of efficient solution of Remote Association Tests

Summary:
The authors report a series of computational simulations on the SWOW free association network to investigate search strategies involved in the solving of Remote Associates Task. They find that the difficulty of the RAT problem is correlated with structure of the test/solution words in the SWOW network, and examined how the mechanisms of solving RATs could be re-interpreted as search algorithms implemented on a network structure.

Overall comments:
I am very supportive of this work as it is a neat demonstration of how psychology can benefit from leveraging both the mathematics of networks and the availability of large-scale behavioral data. The mathematical simulations appear to be properly done; however, I will leave this for reviewers with more specific expertise to comment on the implementation. My comments will focus more on conceptual issues that the authors should address before the manuscript can be accepted.

We are very grateful to the referee for his positive and encouraging comments.

1. More detail about the SWOW network (pp. 3-4). Because the simulations of search strategies are conducted on this network, it is important to provide more details about

the structure of this network and the properties of the dataset used to construct the network. For instance, do the 12k nodes include the responses to the cue words used in the free association task? How were the weights between pairs of nodes determined (e.g., what does w = 1.0 represent in this context)? Table 1 could also include mean in-degree and out-degree.

We added the required information to the first paragraph of the Data Analysis section and Table 1

2. As reviewed in the introduction, there are many possible search strategies that could be implemented to solve the RATs. The authors seemed to have selected a few that made intuitive sense to them to test. My question to the authors is to what extent are these search strategies psychologically plausible given what is known in the psychological literature about problem solving?

In response to this comment we have added a paragraph in the introduction describing different approaches to the nature of creativity.

The overall structure of the search strategies described in "Enhancing the probability..." sections, that is, combination of following sequences of associations and intermittent abrupt returns to the original stimuli words, is in perfect agreement with what is known about mechanisms of RAT solution from psychological literature (see discussion in the Introduction). The more fine details (how often the returns happen, threshold, filtering overstrong associations) are less well-supported, although, e.g, it is known that psychogical processes typically do involve activation thresholds, and there is some evidence in literature that filtering out sterotypical results leads to larger creativity (see new reference [26] and its discussion in the introduction).

3. I did not quite understand how the lack of a correlation between inverse association weight and difficulty of the RAT led to the argument that the problem of solution recognition is not relevant in this situation (p. 6) and how the simulations with the activation threshold led to the conceptual claim that associative strengths of the links modulated the solvability of the RAT (p. 8). It may be simply be that clearer explanations are needed in these sections.

We completely rewrote the paragraph on page 6, which the referee has mentioned. We added two additional paragraphs of qualitative discussion in the subsection about model with a threshold. We hope that the new version of the presentation is easier to follow.

4. Could the authors provide more discussion theoretical and conceptual implications of their findings? For instance, the RAT is commonly used to evaluate creativity levels among people. Do their findings suggest that creativity can be attributed to the use of appropriate search strategies given the hardness of the RAT (a process-based explanation) or due to structural differences in ones mental lexicon structure (as proposed by Kenett et al.)?

It is a very interesting question indeed, we are very happy to discuss it in the discussion section. Given that in this work we only study the aggregate data on association networks and on the hardness of RATs, our results are compatible with both these assumptions about the nature of creativity. Indeed, we show that following moderately weak associations is benefitial for creativity but how exactly do creative people enhance these weak associations - are they intrinsically stronger in creative persons' minds (one may call it a Mednick-Kenett interpretation) or are the creative people able to suppress strong associations at will (call it Benedek-Neubauer interpretation), we cannot say from the aggregate data. It would be very interesting to design experiments to distinguish between these two interpretations.

We added two new paragraphs discussing this topic to the conclusion.

5. Finally, may I suggest the following references to be included in the paper; justification is provided in parentheses.

Siew, C. S. Q., Wulff, D. U., Beckage, N., & Kenett, Y. (2019). Cognitive Network Science: A review of research on cognition through the lens of network representations, processes, and dynamics. Complexity, 1-24.
[this review is viewed as the state of the art in summarizing the literature on cognitive network science]

Monaghan, P., Ormerod, T., & Sio, U. N. (2014). Interactive activation networks for modelling problem solving. In Computational models of cognitive processes: Proceedings of the 13th Neural Computation and Psychology Workshop (pp. 185-195).
[the authors appear to have conducted a similar set of simulations with respect to the RAT; chapter also contains relevant references about the existing literature on problem solving and creativity]

We are grateful to the referee for suggesting these two very relevant references. We have added them to the manuscript.

Reviewer #2: The authors produced an interesting investigation of the mental processes exploring linguistic knowledge in the human mind in order to solve the remote association task (RAT), e.g. a game where a person is given three cues and needs to find a target concept related to all these cues. The authors operationalise mental search through simple Markov chain random walks and more complex activation spreading models over a weighted network of free associations. The authors find activation spreading provides better results than MC walks in efficiently solving the RAT problem, providing additional quantitative evidence for the relevance of activation spreading modelling in lexical processing. The authors also find that weaker free associations are important for solving harder, rather than easier, RAT problems, in line with previous theories about fluid intelligence and creativity. The manuscript is well written, clearly motivated and nicely outlined. The authors have to be praised for their work and transdisciplinary efforts in providing several

modelling approaches to cognitive data. I only have a few minor suggestions that might slightly improve some passages. The manuscript would nicely suit the multidisciplinary audience of PLOS ONE interested in networks and cognitive data. For the above reasons, I recommend publication of this manuscript on PLOS ONE after (very) minor revisions.

We are very grateful to the referee for his positive and encouraging comments.

--- Comments:
Line 120: How did the authors perform the statistical fitting of the degree distribution with a power-law? The authors should report goodness-of-fit values and argument why the power-law distribution is the best fitting model in comparison to other alternatives for heavy-tailed distribution. Notice that this is easy to fix with packages like powerRlaw in R or powerlaw in Python. Notice also that usually the cumulative distribution shows less noise on the tail, making the fitting more robust.

Thanks a lot for information about powerlaw package in Python, we performed fitting with different distributions and added these results as Supplementary Information.

Line 166: I am not sure but is the one in equation 1 really a probability? The strengths are normalised between 0 and 1 or are they simply counting the amount of people who performed that associations (as I guess it is custom in the SWOW dataset?). A minor rewording might help here.

The association strengths can be interpreted as conditional probability of a response given a cue, then the equation 1 can be considered as probability of finding correct answer in one-step strategy. To avoid misunderstanding, we added the relevant explanation to the first paragraph of the Data Analysis section.

Line 268: Please define the model accuracy.

We rewrote the penultimate paragraph of the "Search with an attraction to the stimuli" subsection, and added the explicit definition of model accuracy there.

Discussion: In the Discussion the authors should address a limitation of their approach: that search in semantic memory is driven only by free associations and not by other types of linguistic relationships, e.g. feature sharing, synonyms, etc. This assumption should be stated but also mitigated by the fact that free associations already possess a multilayer nature, as they can be considered not only as coming from memory patterns but also from the other approaches. But would we expect similar results if we used another type of semantic cognitive network?

We are grateful to the referee for this interesting suggestion.

We performed the correlation analysis on different semantic networks (see Supplementary), weak correlation shows the importance of multilayer free-association links. We added a new paragraph to the discussion addressing this question.

```
Discussion: Does the search with an attraction implement any optimization of shortest
path length/strength? This would nicely relate the search strategy implemented by
activation spread to a process of network economy, where mental paths should
not travel around that much on the network as an indirect consequence of activation
spreading across neighbors.
```

The answer is yes, to some extent. As we mention in the text, the problem with the mechanism described in eq. 2 is that, although probabilities of eventually reaching the solution are quite high, and correlate nicely with the experimental hardness, the resulting path lengths can be unrealistically long.

Already eq. 4 is motivated by curtailing unrealistically long paths: introduction of the resetting probability λ is effectively adding a weight λ^N) to each path (here N is the length of the path). As a result, paths much longer than $-1/\ln\lambda$ are effectively suppressed. We added a comment underlying this to the text.

The algorithm with attraction used in the next section is harder to treat analytically, but it is also definitely skewed towards shorter paths due to the fact that the immediate vicinity of the stimuli words by construction always remains part of the search area.

Finally, when studying the behavior of the algorithm under removal of strong links, we observe the decrease of the mean length of solving trajectories. One can think of this as a sort of path optimization. Given that it is coupled with the increase of the accuracy, one can speculate that finding a weak but short path to the solution is more efficient than finding a strong connection via long chain of associations. We included a comment about this into the text.

```
Typos:
Multiplexes -> multiplex networks (notice multilayer networks are more general models
than multiplex networks)
An unified approach > a unified approach
Very week -> very weak
```

We corrected the typos mentioned by the referee.

```
Reviewer #3: The authors present an analysis of the Remote Associations Test (RAT).
Basically, they describe the variance in RAT items (why certain items are more
difficult to solve) using the network/graph of word associations that is based on
a large-scale survey.
Overall, I found their analyses rigorous. The authors use different approaches to
explain why certain test items are solved more easily.
```

We are grateful to the referee for his positive comments.

```
My main issue with the paper
is that it is basically comprised of very sophisticated correlation studies. This is
of course not a bad thing by itself, but it does mean that the conclusions one can
draw from the results about the causal links between what makes RAT items easy
to solve are rather limited. It is my impression that the authors do not communicate
this fundamental limitation well enough, which could lead to false interpretation of
their findings.

The analyses mix between two highly similar questions: 1) Can the solution
word be reached from the stimuli words on the graph?; and 2) Is the location
of the solution word on the graph relative to the stimuli words explain why certain
stimuli words are more difficult to solve than others?

The many good analyses provided in this paper actually answer the first question,
but claim (rather implicitly) to answer the second question. I admit that this is a
fine distinction, but I believe that the authors themselves are aware of it (L196-L209).
```

We respectfully disagree with this comment by the referee. Indeed, the question (1) of the referee is essentially trivial: if stimuli and response belong to the strongly connected component of the association graph (which is indeed the case for all the RATs we studied and the SWOW-EN network) then by definition the response can be reached from the stimuli. It is exactly the question (2) that we are addressing.

Using a variety of search strategies, from mathematically trivial but impractical to more mathematically sophisticated and psychologically realistic, we show that there is indeed a strong correlation between the location of the solution word on the graph relative to the stimuli words and the hardness of corresponding RATs, thus answering the referee's second question in the affirmative.

```
In that sense, the first analysis which computes the aggregated weights as
a correlate of RAT "hardness" is more credible than the later analyses of activation
threshold.

It is clear in these later analyses that "any" word could be reached
(a sort of over-fitting of the model the authors present if you would like), but
the question of how do human participants recognize the solution to be true is
unanswered, and perhaps cannot be answered using these models.
```

We partly agree with the referee on this point. As we mention several times in the text we consider the RAT solution to be a first

passage problem: the participants sequentially consider possible solution words (and of course they can consider any intermediate words while doing that - it is not clear to us why the referee calls this fact "overfitting") until they find a correct response. We assume that participants immediately recognize a correct response as soon as they find it. Also, they never falsely recognize an incorrect response as correct.

This is, of course, a hypothesis, which probably is not always true in real experiment. It is not clear to us how to estimate directly how often this hypothesis fails. However, there are two reasons why we think that the failure to correctly recognize a solution is of relatively small importance compared to the failure to find it. One is anecdotal evidence from our private communications with psychologists who personally performed RAT experiments and who claim, based on their discussions with test subjects, that failures to recognize a correct solution and falsely recognized wrong solutions are relatively rare. Second is the indirect check via inverse association weights (see page 6 of the manuscript). We completely rewrote the corresponding paragraph and hope that it is now easier to follow. But just in case, here is our argument in a nutshell. If failure to recognize correct answer is an important contributor to a solution failure, then it should manifest itself stronger if the connection "response to stimulus" is weak, so RATs with weak inverse links should be, all the rest being equal, harder. We checked that this is not the case and therefore believe that failure to recognize the answer is of minor importance. It is not, in our opinion, a conclusive proof, but it is a strong argument in that direction.

```
I believe the paper would benefit greatly from openly pointing to its own
fundamental limitations, and prefer cautious conclusions rather than strong ones
(e.g., remove L269-L270, but also elsewhere).
```

We rephrased the conclusion to which the referee objects.

```
In addition, the analyses could
have been made much stronger if there was any control or Null model to compare
the results to. For example, if the authors could use a graph/network which is not
based on associations (e.g., from word embedding nearest neighbors,
or words-co-occurrence), and show that these graphs have far smaller correlations
that the association graphs, it would support the superiority of the information
encoded in human associations.
```

We agree with this comment of the referee. We performed the analysis he has suggested and report the results in a new paragraph in the Discussion section, as well as a new Supplementary figure. We added to Supplementary correlation analysis with semantic networks, based on different word embeddings.

```
In general I would say that reporting .7 correlation of the most basic model is
"too high to believe". This remark is not meant to doubt the integrity of
the authors of course. I simply find it hard to believe that a hallmark of human
cognition, RAT that is supposed to capture the ability of problem solving and
creativity, can be predicted to a large extent by aggregated statistics of word
associations. This is after all a psychological variable, and it is very rare
in psychological research to reach such correlations with a single predictor.
 I believe that if the authors could think of this remarkable result more critically,
they would be able to convey their results and conclusions more cautiously
and accurately.
```

We totally agree with the referee that if we claimed that 70% of human-to-human variability in solving a given RAT was explained by some simple model on aggregated associations network, it would have been too improbable to believe. But we claim no such thing.

Indeed, the .7 correlation is the correlation between the position of the words on an aggregated association network and the *average* **hardness of a RAT. It has nothing to do with the human-to-human variability in subjective hardness of a given RAT, but everything to do with RAT-to-RAT variability. That is to say, we are talking about the fact that (i) some RATs are systematically harder than others: indeed, almost for any English-speaking human "cream, skate, water $\to$ ice" is easier than "forward, flush, razor $\to$ straight"; (ii) this big difference between average hardnesses of different RATs is to a large extent explained by the fact that there are strong associations of the type "cream$\to$ice", "water$\to$ice" in the first of the two examples, which are known to almost all English speakers.**

```
Lastly, I find the survey the authors write in L36-L44 interesting, but completely
irrelevant for the rest of the paper. In its current location in the manuscript it
unnecessarily deviates the reader's attention. I would ask the authors to remove it,
change its position, or better explain why it is necessary in the context of this paper.
```

We removed the mention of our unfinished work from this paragraph of the Introduction. However, in our opinion, the fact that word embedding must not nesessarily mean embedding into a Euclidean space is highly relevant to any discussion of the embedding problem. Indeed, the usage of curved spaces allows to reproduce the topology of complex networks using a space with much smaller dimensionality than otherwise needed. This part of introduction is a general overview of different approaches to the study of linguistic networks, including those which are not directly related to the topic of the paper. If the referee thinks such an overview is redundant, we are open to removing the paragraph about word embedding altogether. However, if it remains, we think that it is essential that it mentiones embedding into curved spaces as an important option.

Same goes for the last two paragraphs of the discussion. Although I understand the wish of the authors to promote their own future work (which sounds very interesting), there is no actual link between the current work and their suggested spectral analysis. I would like the authors to either remove these paragraphs, or do a better job in explaining why the discussion of spectral analysis is relevant in the context of the current work (in my opinion it is not).

We accepted this recommendation of the referee and removed the comments concerning the spectral analysis from the discussion section. We will discuss this issue elsewhere.

Typos and Misc.
- The actual RAT items are based on Bowden and Jung-Beeman set, which differs slightly from the original RAT. The authors would do right if they will make this point clearer in the text.

We added the mention of this difference into the text.

L200: the "i" in "invert".
L223: by > be
L305: the "p" in "preferentially"
L346: paople > people
Figure 2: the "hardness" axis is in fact "easyness". The larger values represent easier items.
Figure 3b: I believe the x-axis should be the same as in Figure 2, and not "accuracy".

We have correscted the typos mentioned by the referee.
